# Integrative Bioinformatics Across Proliferative and Non-Proliferative Cell States Reveals Novel Cytokinesis Genes

**DOI:** 10.3390/ijms27198459

**Published:** 2026-09-23

**Authors:** Miriam Angeloni, Marina Leone, Marco Moraschini, Eleonora Lippolis, Felix B. Engel, Fulvia Ferrazzi

**Affiliations:** 1 Institute of Pathology, Friedrich-Alexander-Universität Erlangen-Nürnberg (FAU), 91054 Erlangen, Germany; angeloni.miriam@gmail.com; 2 Comprehensive Cancer Center Erlangen-EMN (CCC ER-EMN), 91054 Erlangen, Germany; felix.engel@uk-erlangen.de; 3 Bavarian Cancer Research Center (BZKF), 91054 Erlangen, Germany; 4 Experimental Renal and Cardiovascular Research, Department of Nephropathology, Institute of Pathology and Department of Cardiology, Friedrich-Alexander-Universität Erlangen-Nürnberg (FAU), Kussmaulallee 12, 91054 Erlangen, Germany; 5 Institute of Human Genetics, Friedrich-Alexander-Universität Erlangen-Nürnberg (FAU), 91054 Erlangen, Germany; 6 Department of Nephropathology, Institute of Pathology, Friedrich-Alexander-Universität Erlangen-Nürnberg (FAU), Krankenhausstr. 8–10, 91054 Erlangen, Germany

**Keywords:** bioinformatics, cytokinesis, heart development, integrative approach, cell cycle, cardiomyocyte

## Abstract

The mammalian heart has no significant endogenous regenerative capacity, as mammalian cardiomyocytes undergo a last round of cell cycle after birth, becoming post-mitotic and polyploid. Consistent with this, cytokinesis inhibition in zebrafish impairs their cardiac regenerative capacity. Notably, re-induction of cell cycle progression or polyploidization reduction through cytokinesis generates proliferative mammalian cardiomyocytes. Thus, a better understanding of cytokinesis is fundamental. Here, we adopted an integrative bioinformatics approach to identify novel common candidate cytokinesis genes by comparing proliferative and non-proliferative cell states. The approach relies on (i) a temporal expression dataset profiling rat ventricles during heart development, (ii) a transcriptomics dataset profiling postnatal rat cardiomyocytes upon stimulation with pro-proliferative compounds, and (iii) an expression dataset profiling mouse embryonic fibroblasts upon knockout of Lin9, a master regulator of cell division. Following prioritization of the identified 236 novel candidate cytokinesis genes and literature-based investigations, *Car8*, *Sox11*, *Arl6ip1*, and *Sc5d* underwent experimental validation. Protein localization studies in HeLa cells revealed distinct localization patterns during cytokinesis for CA8 and SC5D. Their knockdown in Hct116 cells resulted in increased multinucleation. These findings highlight that leveraging integrative bioinformatics across different proliferative states can uncover novel candidate cytokinesis genes, contributing to the establishment of potential therapeutic targets to treat proliferative diseases and/or to promote regeneration.

## 1. Introduction

After birth, the mammalian heart loses its ability to regenerate [1]. One of the causes is the permanent exit of cardiomyocytes from the cell cycle. In rodents, the last round of cell cycle in cardiomyocytes is characterized by mitosis with cytokinesis failure, resulting in binucleated cells. Therefore, rodent hearts consist of more than 90% of binucleated cardiomyocytes. Polyploidization, more than two homologous sets of chromosomes per cell, is a feature of mature cardiomyocytes across mammalian species. In humans, about 40% of cardiomyocytes are tetraploid mononucleated and only about 20% are binucleated [2]. Although the reason for these differences in cellular and nuclear ploidy status across mammalian species is unknown, it has been proven that polyploidization, induced by transient cytokinesis inhibition mediated by dominant-negative Ect2, is a barrier for zebrafish heart regeneration [3]. On the other hand, it has been reported that the reduction in polyploidization through cytokinesis generates proliferative mononucleated cardiomyocytes, resulting in improved ejection fraction after myocardial infarction in murine models [4]. Notably, a recent paper indicates that polyploidization/endoreduplication rates are reduced in patients responsive to a left ventricular assist device, the standard of care for advanced heart failure, which is associated with higher rates of cardiomyocyte renewal (3.1% per year). Finally, studies attempting to re-activate cell cycle progression and cell division to promote cardiomyocyte proliferation-based heart regeneration have revealed that the most critical step is to complete cytokinesis [2,5]. These findings suggest that induction of cytokinesis holds the potential for an attractive future therapy for heart regeneration [6]. Therefore, it is important to understand the mechanism underlying cytokinesis failure in cardiomyocytes. In addition, these mechanisms might be exploited as therapeutical targets for many other diseases such as cancer, psoriasis, and fibroproliferative diseases, in which cell hyperproliferation is a disease driver and thus, inhibition of cytokinesis/cell division is favorable.

In the 90s, it was speculated that the mature sarcomeric apparatus represents a physical impediment for furrow progression [7,8]. Yet, after it has been observed that fetal cardiomyocytes can disassemble their sarcomeric apparatus transiently during native cell division [9], it has also been shown that adult cardiomyocytes can be induced to undergo cytokinesis associated with transient disassembly of the sarcomeric apparatus [10]. Subsequently, the process of cytokinesis failure has been studied more in detail. Fundamental mechanisms of the cytokinetic machinery and many of the proteins involved in cytokinesis are conserved across eukaryotic species [11]. Cytokinesis and chromosome segregation are tightly linked to ensure proper cell division [12]. After nuclear envelope breakdown, chromosomes are aligned at the metaphase plate through microtubules generated from two opposite poles forming a structure called “mitotic spindle”. Once chromosome segregation is initiated, an array of non-centrosomal anti-parallel microtubules, the central spindle, is created between the separating chromosomes [13]. Microtubules of the central spindle as well as astral microtubules emanating from the centrosome provide signals for localized activation of actomyosin ring assembly at the division plane. The anchorage of the constricting actomyosin ring to the cell membrane allows for cleavage furrow formation (telophase), with the final formation of the midbody. Abscission is the last part of cytokinesis and is the physical detachment of the two daughter cells by severing the midbody. Several studies have proven that cardiomyocyte cytokinesis failure is associated with mis-localization of fundamental proteins involved in cytokinesis, such as Anillin, RhoA, and IQGAP3 [14]. Based on these data, it is hypothesized that aberrant mitotic microtubule distribution, together with defective actomyosin ring anchorage to the plasma membrane coupled with cleavage furrow regression, results in cytokinesis failure in cytokinesis [14]. However, few studies have focused on understanding the underlying molecular mechanisms [2,14,15,16,17,18,19,20].

One approach to elucidate molecular mechanisms underlying cellular processes is the integration of datasets describing the cellular process of interest in different contexts. Notably, omics analyses often result in a large number of candidate genes including many false positives, which not only increases the downstream decision-making burden, but it also raises the risk of prioritizing suboptimal targets. Thus, it is essential to devise approaches to identify high-confidence candidates for further experimental validation. Integrative bioinformatics strategies address this challenge by jointly interrogating multiple independent sources of information to identify convergent signals across datasets [21]. By leveraging the concordant outcomes resulting from distinct experimental settings, these methods can substantially reduce noise and improve the reliability of the inferred candidates. Integrative analyses have demonstrated their effectiveness to answer a variety of biological questions [22,23,24], and their potential has grown markedly with the increasing public availability of high throughput datasets [25,26]. A key step in order to extract robust insights is to carefully select the datasets to integrate. Indeed, the intrinsic complexity of biological systems, coupled with technical artifacts and experimental variability, makes biological experiments highly heterogeneous.

Here, we devised an integrative bioinformatics approach to identify potential novel common cytokinesis genes by comparing proliferative and non-proliferative states of cardiomyocytes and mouse embryonic fibroblasts (MEFs) lacking Lin9, a core protein of the DREAM MuvB complex, which plays a key role in mitosis and cytokinesis. For this purpose, we combined (1) the “Heart Development (HD) dataset”, profiling temporal gene expression of rat ventricles from embryonic to postnatal stages [27], leveraging the fact that rat cardiomyocytes lose their ability to proliferate around birth due to cytokinesis failure; (2) the “p38i + FGF1 dataset”, utilizing our finding that stimulation of neonatal rat cardiomyocytes with a p38 mitogen-activated protein kinase inhibitor (p38i) and fibroblast growth factor 1 (FGF1) induces cytokinesis and proliferation [10]; (3) the “Lin9 dataset”, which compares gene expression levels of proliferative MEFs and non-proliferative MEFs, genetically induced by LIN9 knockout (KO) resulting in cytokinesis failure and multinucleation [28]. Potential candidate genes involved in cytokinesis were subsequently validated in two independent, proliferative cell types, HeLa and Hct116 cells.

## 2. Results

### 2.1. Integrative Bioinformatics Analysis Identifies Potential Novel Cytokinesis Genes

In order to identify potential novel cytokinesis genes, we relied on an integrative bioinformatics analysis of three independent transcriptomic datasets that capture established cytokinesis-related gene signatures. First, we analyzed a high-resolution, in-house dataset (the “HD dataset”) covering the temporal gene expression of rat ventricles from embryonic to postnatal stages, for a total of 44 time points [27]. The analysis results from this dataset were combined with two other transcriptomic datasets. The “p38i + FGF1 dataset” profiling P2 rat cardiomyocytes induced to re-enter cell cycle upon stimulation with p38i and FGF1 [10], and the “Lin9 dataset” profiling changes in gene expression levels in *Lin9* KO MEFs [28].

As the aim of our analysis was to focus specifically on previously uncharacterized cytokinesis genes, genes already associated with cytokinesis or cell cycle-related processes were excluded from the integrative analysis. This filtering step was performed using Gene Ontology (GO) annotations to identify the set of genes annotated to cytokinesis- or cell cycle-related terms, hereafter respectively referred to as GO-based cytokinesis-/cell cycle-related genes (Figure 1). This filtering strategy was designed to remove cytokinesis genes that also carry broader cell cycle annotations, thereby enriching the candidate set for genes whose potential involvement in cytokinesis/cell division was not yet represented in the GO. On the basis of 87 GO terms matching the regular expression “cytokine[^\\s-]|m phase”, 326 unique GO-based cytokinesis-related genes were identified (Tables S2 and S3). A list of 6049 unique GO-based cell cycle-related genes was instead derived (Figure 1), starting from 1447 GO terms annotated with one of the following words or regular expression: “cell cycle”, “spindle”, “mitotic”, “cell differentia”, “heart development”, “cell division”, (cytokine[^\s-]), “m phase” (Tables S4 and S5). All the GO-based cytokinesis-related genes were also included in the GO-based cell cycle-related genes.

Following the identification of the GO-based cytokinesis-/cell cycle-related genes, each of the three datasets included in the study was independently analyzed to identify candidate cytokinesis genes not previously annotated with cytokinesis- or cell cycle-related GO terms. Each individual analysis yielded a set of candidates that were then integrated to generate a final list of putative novel cytokinesis genes. The starting point for our integrative analysis was the HD dataset. In the HD dataset, 3349 probe sets, mapping to 3012 unique genes, were identified as differentially expressed (DE) during heart development. These DE genes were significantly enriched in cytokinesis-related processes (*p*-value = 9.24 × 10^−43^). To identify potential novel cytokinesis genes in the DE probe sets, a knowledge-based approach was devised, based on the guilt-by-association principle, i.e., the hypothesis that genes with similar expression patterns are likely to be implicated in the same biological process [29]. We first examined the DE probe sets to identify probe sets targeting genes included in the list of GO-based cytokinesis-related genes. This analysis revealed that 68 DE probe sets targeted 58 unique cytokinesis-related genes; the temporal expression profiles of these probe sets were defined as the “reference profiles” (Figure S1). We then assessed whether any of the remaining DE probe sets, not targeting GO-based cytokinesis-related genes, exhibited expression profiles with high Pearson correlation (i.e., higher than 0.99) with at least one reference profile. The correlation analysis identified 141 probe sets targeting 135 unique genes. After excluding from these genes, the ones included in the GO-based cell cycle-related gene list, a final list of 83 probe sets targeting 81 unique genes was obtained, which we called “knowledge-driven candidate cytokinesis genes” (Table S6 and Figure S2).

After the identification of these candidate cytokinesis genes, we proceeded with the analysis of the p38i + FGF1 dataset. It has been shown that postnatal cardiomyocytes are able to re-enter the cell cycle and undergo cytokinesis resulting in cell division, upon stimulation with p38i and FGF1 [10]. Notably, while p38 inhibition alone upregulated RNA expression of core components of the central spindle [15], it was not sufficient to drive cardiomyocytes into cytokinesis. Thus, we hypothesized that genes strongly expressed in the combined stimulation with p38i and FGF1 (p38i + FGF1), and at the same time less strongly expressed in the stimulation with FGF1 alone and p38i alone, are more likely be the ones associated with the cytokinesis processes. This hypothesis was strengthened by the analysis of the log2FC distributions of the reference profile probe sets from the HD dataset in the p38i + FGF1 dataset across the three conditions (i.e., p38i, FGF1, and p38i + FGF1). This analysis revealed higher log2FCs in the p38i + FGF1 condition compared with FGF1 and p38i (Figure 2). In addition, the analysis validated our previous studies revealing significantly higher log2FC for p38i compared to FGF1 [15]. The thresholds identified on the log2FC distributions for the three conditions and used to select the list of candidate cytokinesis-related genes were thr_p38i_ = 1.305 (corresponding to the 90th percentile), thr_FGF1_ = 0.964 (corresponding to the 90th percentile), and thr_p38i + FGF1_ = 0.663 (corresponding to the 50th percentile) respectively (Figure 2). The threshold thr_p38i + FGF1_ was defined so that the log2FCs_p38i + FGF1_ of the master cytokinesis genes *Anln*, *Iqgap3*, *Ect2*, *Aurkb*, and *Plk1* [30,31] were all above thr_p38i + FGF1_ in both experimental replicates. In contrast, the great majority of these master genes had log2FC_p38i_ and log2FC_FGF1_ lower than thr_p38i_ and thr_FGF1_, respectively. A total of 625 unique probe sets, matching 485 genes, met the required condition, i.e., (log2FC_p38i + FGF1_ > thr_p38i + FGF1_) AND (log2FC_p38_ < thr_p38i_) AND (log2FC_FGF1_ < thr_FGF1_). These 485 genes resulted significantly enriched in cytokinesis-related processes (*p*-value = 1.81 × 10^−8^). In addition, 172 probe sets out of 625 overlapped with the DE probe sets in the HD dataset. These 172 probe sets were associated with 158 genes, which were also significantly enriched in cytokinesis-related processes (*p*-value = 0.04). In order to focus on novel candidate genes, the GO-based cytokinesis-/cell cycle-related genes were removed from the list, thus obtaining 77 unique p38i + FGF1 candidate cytokinesis genes (Figure 3a and Table S7).

After the identification of the p38i + FGF1 candidate cytokinesis genes, we proceeded with the analysis of the Lin9 dataset. Lin9 is a member of the DREAM complex, which has a key role in mitosis progression and cytokinesis by regulating important genes such as *Plk1*, *Aurka*, and *Kif20a* [30]. Therefore, we hypothesized that genes required for cytokinesis would likely be downregulated in the absence of Lin9 in MEFs, while potential cytokinesis inhibitors would be upregulated. In the Lin9 dataset, a total of 3806 spots with a mean FC across the four hybridization replicates bigger than 1.5 or lower than −1.5 were identified. These spots, corresponding to 3792 unique genes, were significantly enriched in cytokinesis-related processes (*p*-value = 6.93 × 10^−10^). A subset of 104 genes was also found among the DE genes of the HD dataset. This subset was not significantly enriched in GO-based cytokinesis-related processes (*p*-value = 0.08). After filtering out from the 104 gene list the GO-based cytokinesis- and cell cycle-related genes, a final list of 94 potential novel cytokinesis genes hereafter referred to as “Lin9 candidate cytokinesis genes” was identified (Figure 3b and Table S8).

The final gene list of 236 unique candidate cytokinesis genes (Table S9) was obtained by integrating the gene lists derived from the individual analyses of the three datasets.

### 2.2. Biological Validation Confirms the Role of Car8 and Sc5d in Cytokinesis

To further prioritize the 236 candidate cytokinesis genes for experimental validation, preference was given to those genes that were supported by more than one independent dataset, reasoning that recurrence across more than one dataset provided stronger evidence than detection in a single dataset. More specifically, priority was given to those genes that (i) were present both in the p38i + FGF1 and in the Lin9 candidate cytokinesis gene lists (and thus were DE in the HD dataset) or (ii) were among the knowledge-driven candidate cytokinesis genes and belonged either to the p38i + FGF1 or to the Lin9 candidate cytokinesis genes. This prioritization step narrowed the initial 236 candidates down to 15 candidates (Table S9), namely *Car8*, identified in all three datasets, and 14 additional genes that were identified in at least two. This list of 15 genes subsequently underwent manual curation to determine whether any genes had already been described as involved in cell cycle or cytokinesis. This step reduced the list to five genes, i.e., *Car8*, *Sc5d*, *Ccser2*, *Arl6ip1*, and *Sox11* (1383210_at, assigned to *Sox11* based on blasting of the target sequence), which, to the best of our knowledge, had not previously shown any association with cell cycle or cytokinesis. In the HD dataset, for *Car8*, a marked expression peak during mid-embryonic stages (E14), followed by a decline toward birth was observed (Figure 4a). This profile is consistent with the temporal window in which cardiomyocytes actively undergo cell division during embryogenesis and subsequently exit cell cycle becoming binucleated in the early postnatal period [32]. *Sc5d*, *Arl6ip1*, and *Sox11* displayed a progressive decrease in their expression across cardiac development (Figure 4b–d), consistent with a profile expected for known positive cytokinesis genes, such as *AurB* and *Anln* [33,34]. Instead, *Ccser2* showed a progressive increase in its expression from embryonic to postnatal stages (Figure S3). Based on the hypothesis that cytokinesis-related genes are expected to be upregulated during the proliferative embryonic phase and downregulated after birth, *Ccser2* was excluded from further validation. Therefore, the final experimental set comprised four genes, i.e., *Car8*, *Sc5d*, *Arl6ip1*, and *Sox11* (1383210_at) (Table S9).

The four final candidates (*Ca8*, *Sc5d*, *Arl6ip1*, and *Sox11*) were validated in vitro and their cellular localization examined during interphase, metaphase, and cytokinesis (Figure 5). The minimum requirement for a protein to be involved in cytokinesis is that its localization is present in at least one of the phases characterizing cytokinesis, e.g., central spindle formation, localization of actomyosin ring assembly at the division plane, anchorage of the actomyosin ring to the cell membrane, midbody formation, and/or abscission. Since cytokinesis is a dynamic process, it might be that a protein is physically present only in one of the aforementioned phases but still plays a fundamental role, such as CHMP4B [35]. HeLa cells were transfected with plasmids enabling the overexpression of the candidate proteins tagged with myc under the control of a ubiquitous CMV promoter. A co-localization of CA8-myc with mitotic spindle microtubules during metaphase and possibly with the midbody microtubules during cytokinesis was observed (Figure 5). SC5D was absent in metaphase, yet in cytokinesis it seemed to be localized around the basis of both midbody arms in the intracellular bridge. ARL6IP1 was absent in metaphase and exhibited no particular localization during cytokinesis, while SOX11 was diffusely expressed both in metaphase and cytokinesis with a possible re-acquisition of nuclear localization during cytokinesis similar to its localization during interphase. Thus, SOX11 was not advanced to following validation experiments since it did not show an evident association with cytokinesis-related structures. Due to their expression levels and localization during cytokinesis, CAR8 and SC5D were further analyzed in Hct116 knockdown studies. In addition, we analyzed also ARL6IP1 as it showed an unspecific but broad localization around the basis of both midbody arms in the intracellular bridge. To this aim, siRNAs against these targets were utilized, under the assumption that the absence of a cytokinesis gene would cause cytokinesis failure, thus resulting in bi/multinucleation. After validation of the knockdown strategy via qRT-PCR (Figure 6a), the quantity of multinucleated cells in Hct116 cells treated with either siRNA control (ctrl.) or siRNA against the targets was quantified (Figure 6b,c). Notably, knockdown of CA8 and SC5D resulted in a significantly higher percentage of multinucleated cells compared to siRNA ctrl., thus strengthening the potential novel role of these two genes as cytokinesis genes.

## 3. Discussion

During mammalian heart development, the heart grows through cardiomyocyte proliferation, a process that rapidly ceases soon after birth. The adult mammalian heart has no, or very limited, capacity to regenerate, which has been widely associated with the permanent withdrawal of cardiomyocytes from the cell cycle. Understanding the molecular mechanisms underlying this transition is crucial for identifying new therapeutic strategies aimed at reactivating cardiomyocyte proliferation in the adult heart. It has been shown that cardiomyocytes can be relatively efficiently induced to re-enter the cell cycle. The major hurdle to overcome is to promote efficient cytokinesis resulting in cell division. In this work, we therefore performed an integrative bioinformatics analysis involving three independent transcriptomics datasets to identify potential novel cytokinesis genes. The HD dataset, which contains high-resolution gene expression profiles of rat ventricles measured across multiple developmental stages, served as our primary model to study proliferation-related expression changes [27]. It allowed for us to explore how cytokinesis-related genes are regulated during heart development and cardiomyocyte maturation and to identify patterns associated with the loss of proliferative potential after birth. Based on the “guilt by association” principle, genes with expression profiles similar to known cytokinesis genes were hypothesized to share related biological functions.

To enhance the robustness of the analysis and minimize dataset-specific biases, we complemented the HD dataset analysis with two independent datasets, namely the p38i + FGF1 dataset [10] and the Lin9 dataset [28]. The choice of these two datasets was driven by their biological relevance to cell cycle control and cytokinesis reactivation. Notably, the p38i + FGF1 dataset allowed for us to identify genes whose expression responds to conditions that promote cytokinesis. The Lin9 dataset, on the other hand, provides an independent system in which perturbation of *Lin9*, a member of the DREAM complex, a well-established cytokinesis regulator, inhibits mitotic progression and cytokinesis.

By integrating the results of these three datasets, we were able to identify a final list of potential novel candidate cytokinesis genes shared across independent studies, thereby increasing the reliability of our findings. It is worth noting that the goal of our integrative bioinformatics analysis was to prioritize candidate cytokinesis genes for experimental validation. Thus, changes in some analysis parameters, such as relaxing the high correlation threshold utilized to identify similarity with the reference profiles, might lead to a larger candidate list to be investigated in the future. Our experimental validation strengthened the hypothesis that *Car8* and *Sc5d* encode new cytokinesis genes. CA8 encodes carbonic anhydrase VIII, but unlike classic carbonic anhydrases it does not appear to have strong CO_2_-hydration enzyme activity [36,37]. The exact biological function of CA8 remains elusive whereby it appears to have a major function in controlling intracellular calcium [38]. CA8 is strongly expressed in the cytoplasm of Purkinje cells of the cerebellum [39]. In the context of proliferation, it has been positively associated with a variety of tumors, such as ovarian carcinomas [40] as well as colorectal cancers [41]. Nishikata and colleagues showed that CA8 is a positive regulator of colon cancer growth by regulating cellular processes such as cell proliferation, colony formation, and cell invasion [42]. However, the authors didn’t dissect the importance of CA8 in specific cell cycle/mitosis phases. SC5D is an enzyme of the cholesterol biosynthesis and its role in cell cycle is not well characterized. Based on The Human Protein Atlas, SC5D is mainly localized in the nucleoplasm and endoplasmic reticulum and, based on the cell cycle reporter FUCCI, SC5D expression does not correlate with the cell cycle. A recent publication showed that upon SC5D knockout liver cells have a slower rate of proliferation compared to the control cells due to elevated lathosterol; more specifically these cells are characterized by an increased G0-G1 population with a respective reduction of S and G2/M cell populations [43]. Notably, this phenotype was only observed when lipid depleted serum was utilized, but not in the presence of fetal bovine serum as used in our study. The localization of SC5D at the basis of both midbody arms is unusual and there is to our knowledge no other protein described with this specific localization. During abscission, the two daughter cells are finally separated by severing the intercellular bridge that remains after the contractile ring has constricted. This is an active membrane-remodeling process, not just a simple cut, and it is tightly controlled by the abscission checkpoint, so the cell does not separate if chromatin is still trapped in the bridge [44]. Cholesterol appears to be important for this stage, especially for local endocytic membrane remodeling at the intercellular bridge. In one study, blocking cholesterol-dependent endocytosis reduced the formation of midbody tubules and caused multinucleation, indicating that cholesterol-rich membrane dynamics contribute to successful cytokinesis [45].

Here, we have preliminarily assessed the possible role of CA8 and SC5D in cytokinesis via overexpression of myc-tagged proteins under the control of a CMV promoter and the endpoint readout “multinucleation” upon gene depletion. Both used experimental approaches were based on still photos of immunostainings lacking a deep analysis of the mechanistic role of these genes in cytokinesis. Thus, future studies are required to improve the knowledge around CA8 and SC5D. Of note, the use of a CMV promoter can potentially alter normal intracellular localization of the analyzed proteins. Live-cell time-lapse imaging to quantify chromosome segregation, furrow ingression, regression, and bridge duration utilizing inducible knockdown cells will reveal which step in mitosis or cytokinesis is impaired. In addition, cell lines should be imaged in which endogenous CA8 and SC5D is fluorescently labeled (knock-in). These studies should be combined with an immunofluorescence staining analysis for markers of the furrow (F-actin, myosin II, anillin), midbody (MKLP1, PRC1, Cep55, Aurora B), and abscission (CHMP2A, ALIX, TSG101) [46,47,48,49] in order to determine possible mislocalization of these fundamental cytokinesis proteins.

Our candidate selection strategy was intentionally focused on genes showing high expression during embryonic cardiomyocyte proliferation and downregulation after birth. It is worth underlying, though, that while this criterion effectively enriches for putative genes required for cytokinesis, it may overlook genes with inhibitory roles. Potential negative regulators or inhibitory pathways of cytokinesis might exhibit the opposite developmental trajectory, i.e., low expression during embryogenesis that steadily increases towards the postnatal stage during heart development. As a result, our approach provides a focused but necessarily partial view of the broader and complex regulatory network controlling cytokinesis. Of note, it is important to add that pharmacological inhibition approaches have higher success rate in entering the market for a therapeutical vision. Importantly, inhibiting cytokinesis by one gene appears a promising strategy to block or delay proliferation in proliferative diseases. In contrast, induction of cytokinesis by activation a single gene appears unlikely to promote cell proliferation and regeneration. Notably, it has recently been reported that the transient induction of two cytokinesis genes generates highly proliferative mononuclear cardiomyocytes [4]. More promising for a cardiac therapeutical prospective appears to be the identification and inhibition of a potential cytokinesis inhibitor. However, it is currently unclear whether cardiomyocyte proliferation stops after birth only because certain cell cycle genes are inactive or due to an active mechanism repressing cytokinesis. Thus, future unbiased or complementary screening strategies will be required to capture the full repertoire of both positive and negative cytokinesis regulators.

## 4. Materials and Methods

All data analyses were performed in R v. 4.5.1 [50].

### 4.1. Identification of GO-Based Cytokinesis-Related Genes

To identify novel cytokinesis genes in heart tissue, the first step relied on the extraction of a list of genes already known to be associated with cytokinesis-related processes. To identify this gene list, we first retrieved from the Gene Ontology (GO) [51,52] all the GO terms, with the respective children, related to cytokinesis processes. We then retrieved, for each cytokinesis-related term, the associated set of genes based on the gene annotation file. The list of unique genes belonging to the identified GO terms was taken as reference gene list of known cytokinesis genes. More specifically, first the whole GO was downloaded in OBO (go.obo) file format (https://geneontology.org/docs/download-ontology/ (accessed on 3 April 2025)) [53] and read in R using the function get_ontology() from the R package ontologyIndex v2.12 [54]. The function was run with the argument extract_tags = “everything”, to extract all properties provided by the OBO file, and specifying for the argument propagate_relationships the following strings: is_a, part_of, regulates, negative_regulates, positively_regulates, occurs_in. All the GO terms whose names matched the regular expression “cytokine[^\\s-]|m phase” (e.g., “mitotic M phase”, “regulation of cytokinetic process”) were identified as cytokinesis-related terms and referred to as “primary matches”. Next, for each primary match, the associated child term(s) were retrieved (hereafter referred to as “secondary matches”). The final list of cytokinesis-related GO terms was defined as the union of primary and secondary matches after removal of duplicate terms. To assign the respective set of genes to the final list of cytokinesis-related GO terms, the GO annotation file for Rattus norvegicus (rgd.gaf)—release version 2025-03-16 (https://current.geneontology.org/products/pages/downloads.html (accessed on 3 April 2025)) —was used. The annotation file was imported in R relying on the function readGAF() from the package mgsa [55]. The function was run specifying the arguments “evidence=NULL” to return annotations from all evidence codes, and “aspect=c(“P”, “F”, “C”)” to return annotations from all three ontologies, i.e., biological process, cellular component, and molecular function. The final list of known cytokinesis genes was taken as the union of the genes assigned to the final list of cytokinesis-related GO terms after removal of duplicate genes. Hereafter, we will refer to this set of genes as “GO-based cytokinesis-related genes”.

### 4.2. Identification of GO-Based Cell Cycle-Related Genes

To filter out from the lists of candidate novel cytokinesis genes those already known to be related to cell cycle processes, a list of genes involved in cell cycle was identified following an analogous strategy to the one described in Section 4.1. Briefly, we first identified all GO terms (“primary matches”) annotated with one of the following words or regular expressions: “cell cycle”, “spindle”, “mitotic”, “cell differentia”, “heart development”, “cell division”, (cytokine[^\s-]), “m phase”. We then retrieved the associated child term(s) (“secondary matches”) and created the final list of cell cycle related GO terms as union of primary and secondary matches after removal of any duplicate terms. Finally, the unique genes associated with the identified cell cycle-related GO terms were extracted and referred to as “GO-based cell cycle-related genes”.

### 4.3. The Heart Development Dataset

The heart development dataset (hereafter referred to as “HD dataset”) contains the gene expression levels of rat cardiac ventricles from embryonic (E) day 11 to postnatal (P) day 10.5 at 12 h intervals, for a total of 44 time points [27]. Expression analysis was performed using the Affymetrix GeneChip RAT 230 Expression Set (chip A and chip B) (Termo Fisher Scientific, Waltham, MA, USA). Data pre-processing, i.e., background subtraction, normalization and probe set summarization, was performed according to the robust multi-array average procedure [56].

To annotate probe sets to target genes, the Affymetrix RAE230 annotation files version na36 (April 2016) for chip A and B were used (https://www.thermofisher.com/de/de/home/life-science/microarray-analysis/microarray-data-analysis/genechip-array-annotation-files.html (accessed on 26 February 2025)). On Affymetrix gene expression microarrays, one probe set can target multiple genes and a gene may be associated to multiple probes. A total of 31,256 probe sets (unique: 31,099) are present on the Affymetrix GeneChip RAT 230 Expression Set, distributed across chip A and chip B. Chip A contains 15,923 probe sets, mapping to 12,027 unique gene symbols, and chip B contains 15,333 probe sets mapping to 7334 unique gene symbols. Chip A and B have 157 probe sets in common, mapping to 114 unique gene symbols. For probe sets targeting multiple genes, all the mapping genes provided in the annotation file were kept to avoid missing any potential gene identifier belonging to the list of known cytokinesis-/cell cycle-related genes.

Differentially expressed (DE) probe sets were identified through an area-based method [57]. In particular, DE probe sets were considered those showing an expression profile markedly different from a constant profile, equal to the value at its first time point.

To identify potential novel cytokinesis genes, DE probe sets mapping to genes belonging to the list of GO-based cytokinesis-related genes were identified. These probe sets will be referred to as “reference profiles”. A pairwise correlation analysis between the reference profiles and the remaining DE probe sets was then performed. The probe sets with expression profiles with a Pearson’s correlation coefficient higher than 0.99 with at least one reference profile and not mapping to any GO-based cell cycle-related gene were considered “knowledge-driven candidate cytokinesis genes”. This stringent threshold was chosen to prioritize genes displaying expression profiles very closely matching those of established cytokinesis genes.

### 4.4. The p38i + FGF1 Dataset

The “p38i + FGF1 dataset” contains the gene expression levels of postnatal day 2 (P2) rat cardiomyocytes upon stimulation with the p38 inhibitor SB203580 (p38i) and FGF1 [10,15]. More specifically, gene expression levels were measured under four different conditions: (i) control condition (DSMO), (ii) stimulation with FGF1 only, (iii) stimulation with p38i only, (iv) combined stimulation with FGF1 and p38i (p38i + FGF1) [10]. Two independent experiments of all 4 conditions in parallel were performed. Analogously to the HD dataset, expression analysis was performed using the Affymetrix GeneChip RAT 230 Expression Set (chip A and chip B). Data were pre-processed as previously described [15]. To identify potential novel candidate cytokinesis genes, first the log2 fold changes (log2FCs) between each treatment condition (i.e., p38i, FGF1, and p38i + FGF1) and DSMO were calculated for the two independent experiments. Hereafter we will refer to these log2FCs as log2FC_p38i_, log2FC_FGF1_, and log2FC_p38i + FGF1_ respectively. A gene was considered a potential novel cytokinesis gene if its matching probe set had a log2FC_p38i + FGF1_ higher than a pre-defined threshold thr_p38i + FGF1_ and log2FC_p38i_ and log2FC_FGF1_, respectively, lower than pre-defined thresholds thr_p38i_ and thr_FGF1_ in both experiments. To summarize, the following condition had to be met for both experiments: (log2FC_p38i + FGF1_ > thr_p38i + FGF1_) AND (log2FC_p38i_ < thr_p38i_) AND (log2FC_FGF1_ < thr_FGF1_). The thresholds thr_p38i + FGF1_, thr_p38i_, and thr_FGF1_ were empirically identified by taking into account the combined log2FC distribution of the reference profiles across both experiments. More specifically, thr_p38i + FGF1_ was defined as the 50th percentile of the combined log2FC_p38i + FGF1_ distribution, as this was the highest percentile threshold for which the condition (log2FC_p38i + FGF1_ > thr_p38i + FGF1_) was satisfied for five well-established cytokinesis regulators, namely *Anln*, *Iqgap3*, *Ect2*, *Aurkb*, and *Plk1*. Instead, thr_p38i_ and thr_FGF1_ were respectively defined as the 90th percentiles of the combined log2FC_p38i_ and log2FC_FGF1_ distributions, on the basis of the hypothesis that candidate cytokinesis genes are more strongly expressed in the combined stimulation with p38i and FGF1 than in the stimulation with FGF1 alone and p38i alone. A gene was considered a potential novel cytokinesis gene in the p38i + FGF1 dataset if (i) it mapped to a probe set meeting the aforementioned condition, (ii) was also present among the DE probe sets of the HD dataset, and (iii) did not belong to the list of GO-based cytokinesis-/cell cycle-related genes. Hereafter we will refer to this set of genes as to “p38i + FGF1 candidate cytokinesis genes”.

Differences between the log2FC distributions of the reference profile genes in the p38i + FGF1 dataset across the three conditions (i.e., p38i, FGF1, and p38i + FGF1) were assessed relying on the Kruskal–Wallis test. Dunn’s test was employed as post hoc test to test for pairwise differences, using Bonferroni correction for *p*-value adjustment. An adjusted *p*-value < 0.05 was considered statistically significant.

### 4.5. The Lin9 Dataset

The “Lin9 dataset” contains gene expression levels of MEFs in four wild type (WT) and four *Lin9* KO samples (ArrayExpress accession number: E-MEXP-2097) [28]. Expression analysis was performed using the Agilent Two-Color Microarray (Agilent Technologies, Santa Clara, CA, USA). To annotate spots to target genes, the Agilent-014868 Whole Mouse Genome Microarray 4 × 44K 014868 (catalog number G4122F) annotation file was used (https://www.ncbi.nlm.nih.gov/geo/query/acc.cgi?acc=GPL4134 (accessed on 20 August 2025)). The annotation file contains 41,174 unique spots, mapping to 21,609 unique gene symbols. To identify candidate novel cytokinesis genes in the Lin9 dataset, the MAGETAB file “NormData_Nina_FGEM.txt.magetab” was downloaded from ArrayExpress (https://ftp.ebi.ac.uk/biostudies/fire/E-MEXP-/097/E-MEXP-2097/Files/NormData_Nina_FGEM.txt.magetab (accessed on 20 August 2025)). The MAGETAB file provides pre-processed data as log2-scale ratios *Lin9* KO vs. WT for four replicate hybridizations (one hybridization for each *Lin9* KO-WT pair), corresponding to two sets of dye-swap experiments. On hybridized arrays 1_1 and 1_2 *Lin9* KO RNA was labelled with green (Cy3) dye and WT RNA was labelled with red (Cy5) dye. On hybridized arrays 1_3 and 1_4, the labelling was the other way around, i.e., *Lin9* KO RNA was labelled with red (Cy5) dye and WT RNA was labelled with green (Cy3) dye. To correct the ratios *Lin9* KO vs. WT for dye effect, the first two columns of the MAGETAB file, corresponding to the pre-processed data of hybridized arrays 1_1 and 1_2, where changed in sign before any further processing. Spots with more than two failed reads (empty value in the MAGETAB file) over the four replicate hybridizations were removed. In case of a gene symbol targeting multiple spots, the spot with the lowest coefficient of variation was assigned to the gene (Table S1). To be able to compare the Lin9 dataset with the HD and p38i + FGF1 datasets, we used the 1:1 mapping gene symbol:spot to search for the orthologue rat gene symbol. More specifically, the mouse gene symbols were provided in input to the function convert_othologues() of the R package orthogene v1.14.01. The function was run with the arguments: input_species = “mmusculus”, output_species = “rnorvegicus”, gene_input = “GENE”, gene_output = “dict”, and non121_strategy = “keep_both_species” to keep all genes regardless of whether they had duplicate mappings in either species. For 14,244 (unique: 12,764) mouse gene symbols, an orthologue rat gene symbol was found (Table S1). Potential novel candidate cytokinesis genes were identified on the processed MAGETAB file as genes (i) showing a mean FC between KO and WT across the four replicate hybridizations higher than 1.5 or lower than −1.5, which corresponds to having the absolute value of the row mean higher than log2(1.5) in log2 scale; (ii) mapping to a DE probe set of the HD dataset; and (iii) not belonging to the list of GO-based cytokinesis-/cell cycle-related genes. Hereafter, we will refer to these candidate genes as to “Lin9 candidate cytokinesis genes”.

### 4.6. Functional Enrichment Analysis of Gene Lists

In order to investigate whether a gene list is enriched for cytokinesis-related processes, the EASE score [58], a modified Fisher’s exact test, as implemented in the DAVID online software [59], was utilized. The EASE score implements a conservative adjustment that, by removing one gene from the list of annotated genes, penalizes the significance of terms supported by few genes. More specifically, a background gene list [population total (PT)] was defined by taking the unique gene symbols on chips A and B (*n* = 15,407). The GO-based cytokinesis-related genes were used as gene list annotated to cytokinesis-related pathways [list total (LT)]. For a given list of genes, the associated list hits (LH) and population hits (PH) were extracted, and the EASE score was calculated relying on a one-sided Fisher’s exact test [R function fisher.test(alternative=‘greater’)] as detailed in the DAVID documentation (https://davidbioinformatics.nih.gov/helps/functional_annotation.html (accessed on 20 August 2025)). If LH = 1, i.e., only one gene in the gene list is annotated to cytokinesis-related processes, the EASE score is automatically set to 1, whereas if LH = 0 then the EASE score is not calculated at all. A gene list was considered enriched with cytokinesis-related processes if its EASE score was below 0.05.

### 4.7. Tissue Culture for the Experimental Validation of Candidate Novel Cytokinesis Genes

HeLa and Hct116 cell line were cultivated in Dulbecco’s Modified Eagle’s Medium-GlutaMAX^TM^ (DMEM-GlutaMAX^TM^) (Invitrogen, Darmstadt, Germany) supplemented with 10% Fetal Bovine Serum (FBS) (Biowest, Nuaillé, France), 100 U/mL penicillin, and 100 µg/mL streptomycin (Sigma-Aldrich, St. Louis, MO, USA). HeLa cell line was a gift from Prof. Gaubatz (Würzburg, Germany), while the Hct116 cell line was a gift from Prof. Stürzl (Erlangen, Germany). All cell lines were grown at 37 °C in a humidified atmosphere of 5% CO_2_.

### 4.8. Plasmids, siRNA and Transfections

All plasmids were purchased from OriGene (Rockville, MD, USA). The human cDNA from each gene was cloned into pCMV6-Entry expression vector. These plasmids allow for the expression of the candidate gene under control of a CMV promoter whereby the candidate gene is tagged with myc, which allows for its detection with an anti-myc antibody. The following siRNAs were purchased from Ambion (Thermo Fisher Scientific, Waltham, MA, USA): siCA8, 5′-CAAUGAUGGACAUACCAUUtt-3′; siSC5D, 5′-GGUUAAUAUCUGGACAAUUtt-3′; siCtrl, Silencer™ select negative control n0.1 siRNA (4,390,843, Thermo Fisher). Plasmids (0.5 µg) were transfected in 24 well (120 000 cells/well) with Lipofectamine^®^ LTX Reagent with PLUS™ (2 µL per well, Life Technologies, Carlsbad, CA, USA). siRNAs (final concentration per siRNA: 65 µM) were transfected in 6 wells (1 × 10^6^ cells/well) with Lipofectamine^®^ RNAiMAX Transfection Reagent (5 µL per well, Life Technologies). After 72 h from siRNA transfection, RNA samples were collected for measuring knockdown efficiency by qRT-PCR and cells were fixed for immunofluorescence analysis.

### 4.9. qRT-PCR

Total RNA was isolated using the Qiagen RNeasy Plus mini kit (74,134, Düsseldorf, Germany), reverse-transcribed (1 µg per reaction) with M-MLV-RT (Sigma, St. Louis, MO, USA) and analyzed with quantitative real-time PCR with SYBER Green reagents from Bio-Rad (Hercules, CA, USA) using the CFX Connect™ Real-Time PCR (Bio-Rad) detection system. Expression differences were calculated relative to β2m. Primer sequences for β2m (forward: 5′-TGACTTTGTCACAGCCCAAGATA-3′, reverse: 5′-AATC CAAATGCGGCATCTTC-3′) were kindly provided by Gelse K. et al. [60].

### 4.10. Antibodies

The following antibodies were used: anti-α-tubulin (1:500, ab6160, Abcam, Cambridge, UK), anti-β-catenin (1:100, 610,153, BD Bioscience, Heidelberg, Germany), anti-myc (1.500, 9B11, Cell Signaling, Cambridge, UK). Primary antibodies were detected using ALEXA 488-, ALEXA 594- or ALEXA 647-conjugated antibodies (1:500, Life Technologies, Carlsbad, CA, USA).

### 4.11. Immunofluorescence Analysis

Cells were cultivated on glass coverslips (CB00120RA1, Thermo Fisher Scientific, Waltham, MA, USA) and fixed for 15 min at room temperature (RT) with 4% formalin/1x PBS. Fixed cells were permeabilized for 10 min at RT with 0.5% Triton X-100/1x PBS and incubated for 20 min at RT with 5% BSA/0.2% Tween 20/1x PBS (blocking buffer). Afterwards, coverslips were incubated with primary antibody diluted in blocking buffer for 1 h at RT, washed three times with 0.1% NP-40/1x PBS, incubated with secondary antibodies diluted in blocking buffer for 45 min at RT, and washed three times with 0.1% NP-40/1x PBS. Nuclei were visualized with DAPI (4′,6′-diamidino-2-phenylindole, 0.5 µg/mL/1x PBS, Sigma) for 10 min at RT. Afterwards, coverslips were mounted on a microscope slide with self-made mounting medium [61].

### 4.12. Statistical Analysis of the Experimental Validation Data

Data are presented as mean ± standard deviation (SD) across at least three independent experiments. Differences between two conditions were assessed relying on a two-tailed Student’s t-test (Microsoft Excel), while one-way ANOVA, as implemented in GraphPad Prism v. 9, was used to compare multiple conditions. A *p*-value ≤ 0.05 was considered statistically significant.

## 5. Conclusions

Collectively, our study demonstrates the power of integrative bioinformatics analyses to uncover novel and biologically relevant cytokinesis genes from transcriptomics data. The identification and experimental validation of new candidate cytokinesis genes provide novel potential targets for the development of new strategies to treat proliferative diseases, such as cancer, idiopathic pulmonary fibrosis, or scleroderma, and/or to promote regeneration. In the latter case this includes understanding the molecular barriers to cardiomyocyte proliferation and developing potential strategies to overcome them thereby promoting heart regeneration.

## Figures and Tables

**Figure 1:**
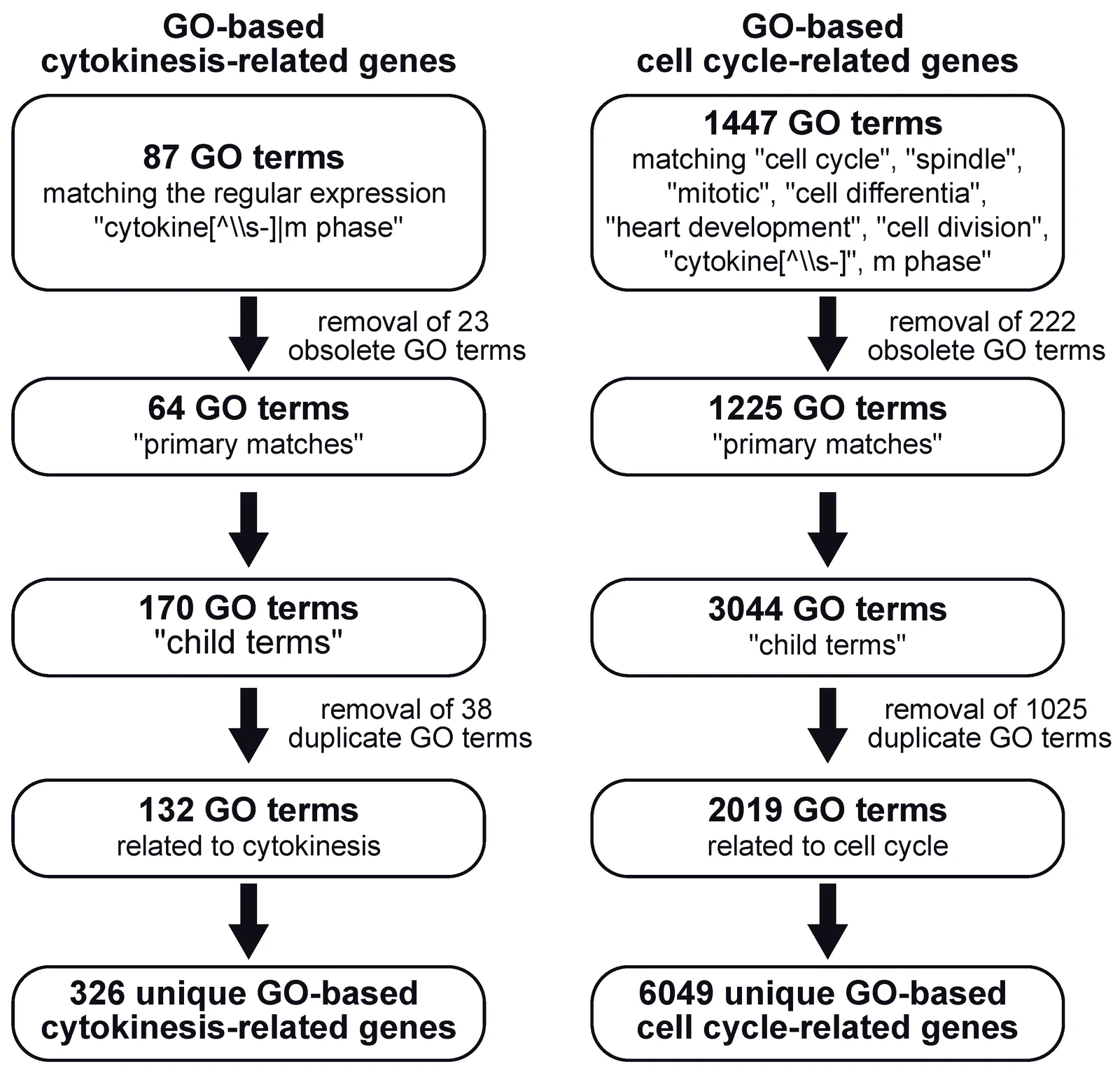
GO-based identification of cytokinesis- and cell cycle-related genes. Flowchart illustrating the Gene Ontology (GO)-based strategy used to define cytokinesis- and cell cycle–related genes. (**left**) For cytokinesis-related genes, 87 GO terms matching the regular expression “cytokine[^\\s-]|m phase” were retrieved. After removal of obsolete terms, expansion to child terms, and elimination of duplicates, 132 unique GO terms related to cytokinesis were retained, associated with 326 unique GO-based cytokinesis-related genes. (**right**) For cell cycle-related genes, 1447 GO terms matching the keywords “cell cycle”, “spindle”, “mitotic”, “cell differentia”, “heart development”, “cell division”, “cytokine[^\\s-]”, and m phase” were identified. Following removal of obsolete terms, inclusion of child terms, and elimination of duplicates, 2019 GO terms related to cell cycle were retained, corresponding to 6049 unique GO-based cell cycle-related genes.

**Figure 2:**
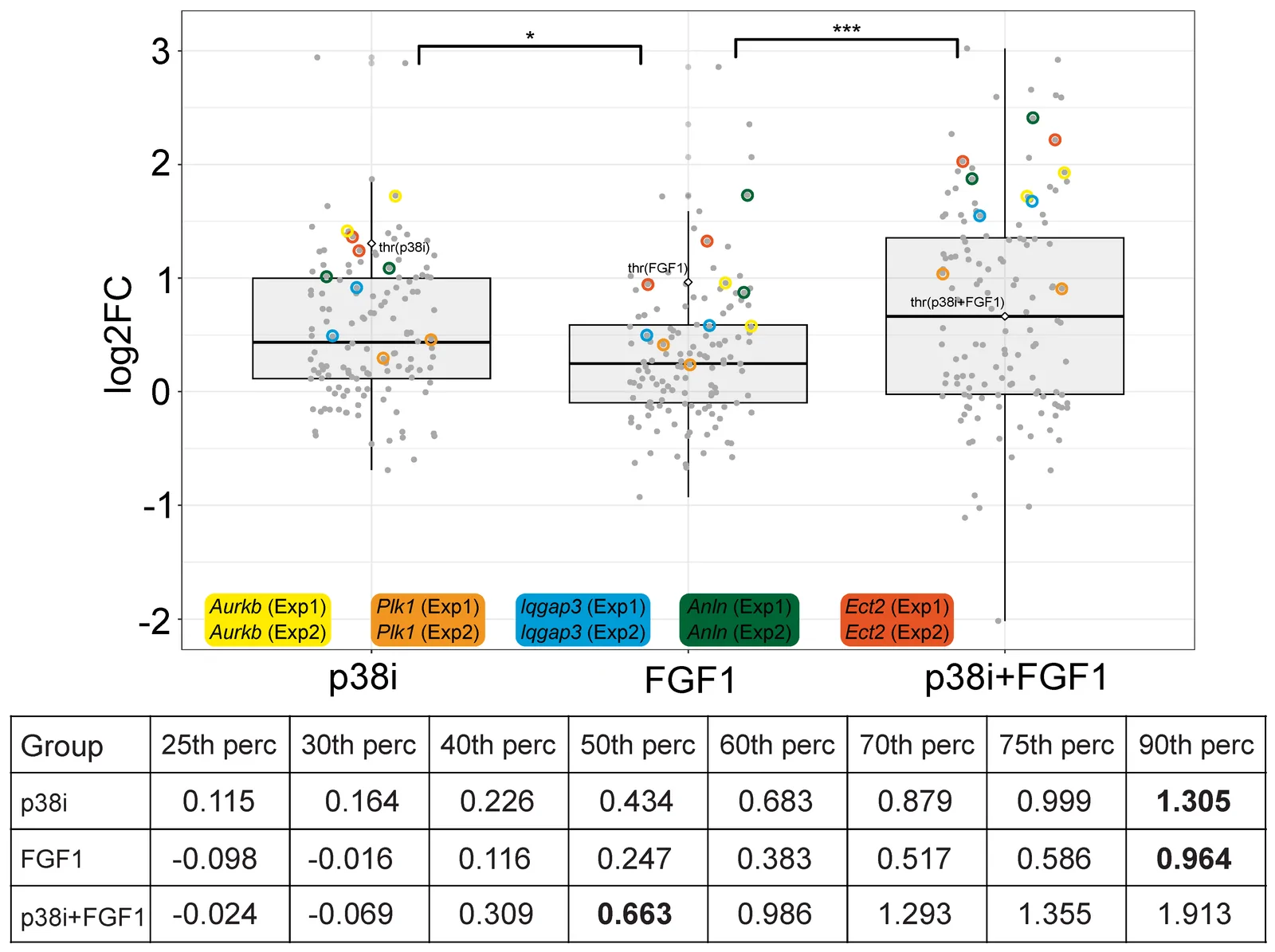
Expression fold changes in the p38i + FGF1 dataset. Boxplot distributions of base-2 logarithms of the fold changes (log2FC) of the reference profile genes in the p38i + FGF1 dataset across the three conditions (i.e., p38i, FGF1, and p38i + FGF1) and the two replicates (Exp1 and Exp2) per condition. For each condition, the identified log2FC thresholds are shown as grey rhombus symbol. The log2FCs of master cytokinesis genes are labeled with colored squared labels as indicated. *: adjusted *p*-value < 0.05, ***: adjusted *p*-value < 0.0005. The table below the boxplots reports the percentiles of the log2FC distributions in the three groups. The thresholds chosen for each group are highlighted in bold.

**Figure 3:**
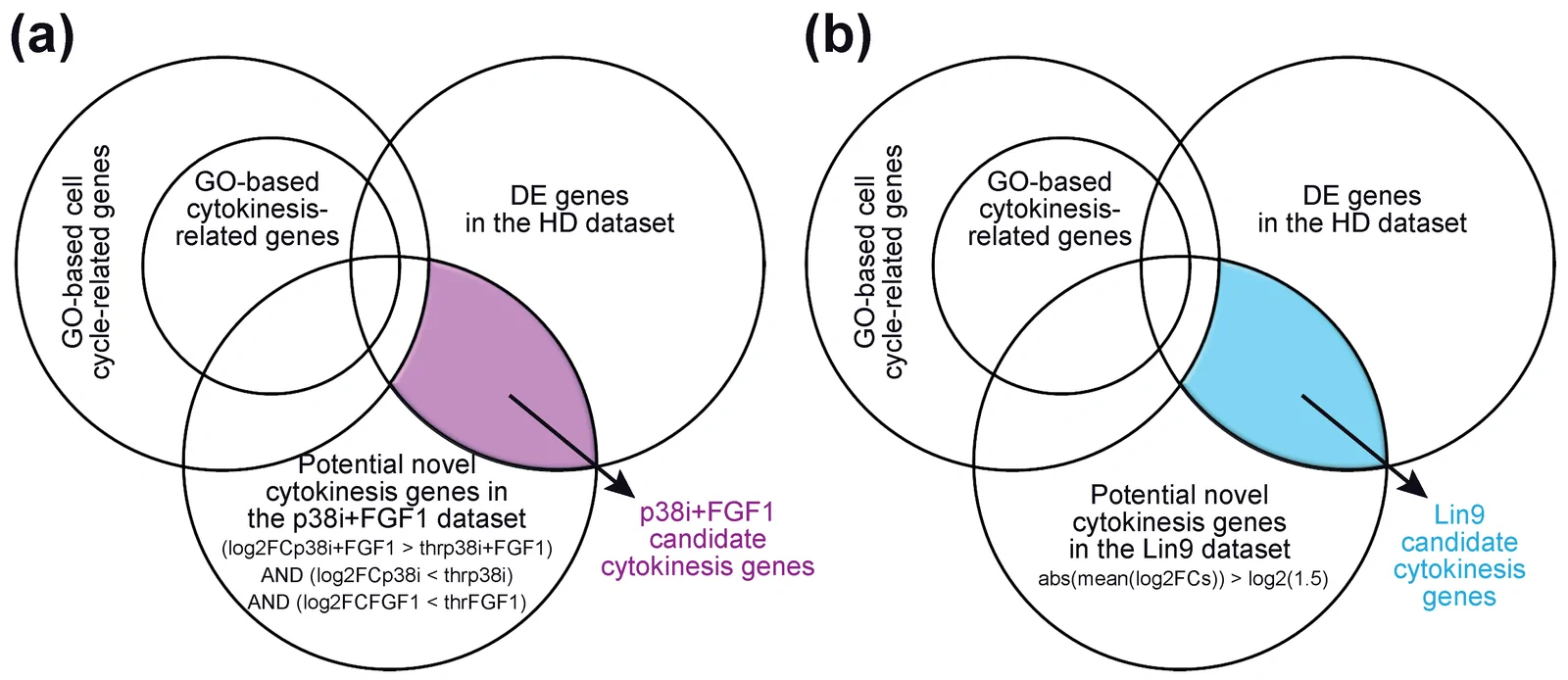
Schematization of the integrative bioinformatics approach. (**a**,**b**) Schematic visualization through Venn diagrams of the strategy utilized to identify candidate cytokinesis genes in the p38i + FGF1 and Lin9 datasets. (**a**) In the p38i + FGF1 dataset, candidate cytokinesis genes (violet) are obtained by intersecting the potential novel cytokinesis genes with log2 fold changes (log2FC) satisfying the indicated conditions with the DE genes in the HD dataset, after removing the GO-based cytokinesis-/cell cycle-related genes. (**b**) In the Lin9 dataset, candidate cytokinesis genes (light blue) are obtained by intersecting the Lin9 potential novel cytokinesis genes with the DE genes in the HD dataset, after removing the GO-based cytokinesis-/cell cycle-related genes. DE: differentially expressed; HD: heart development.

**Figure 4:**
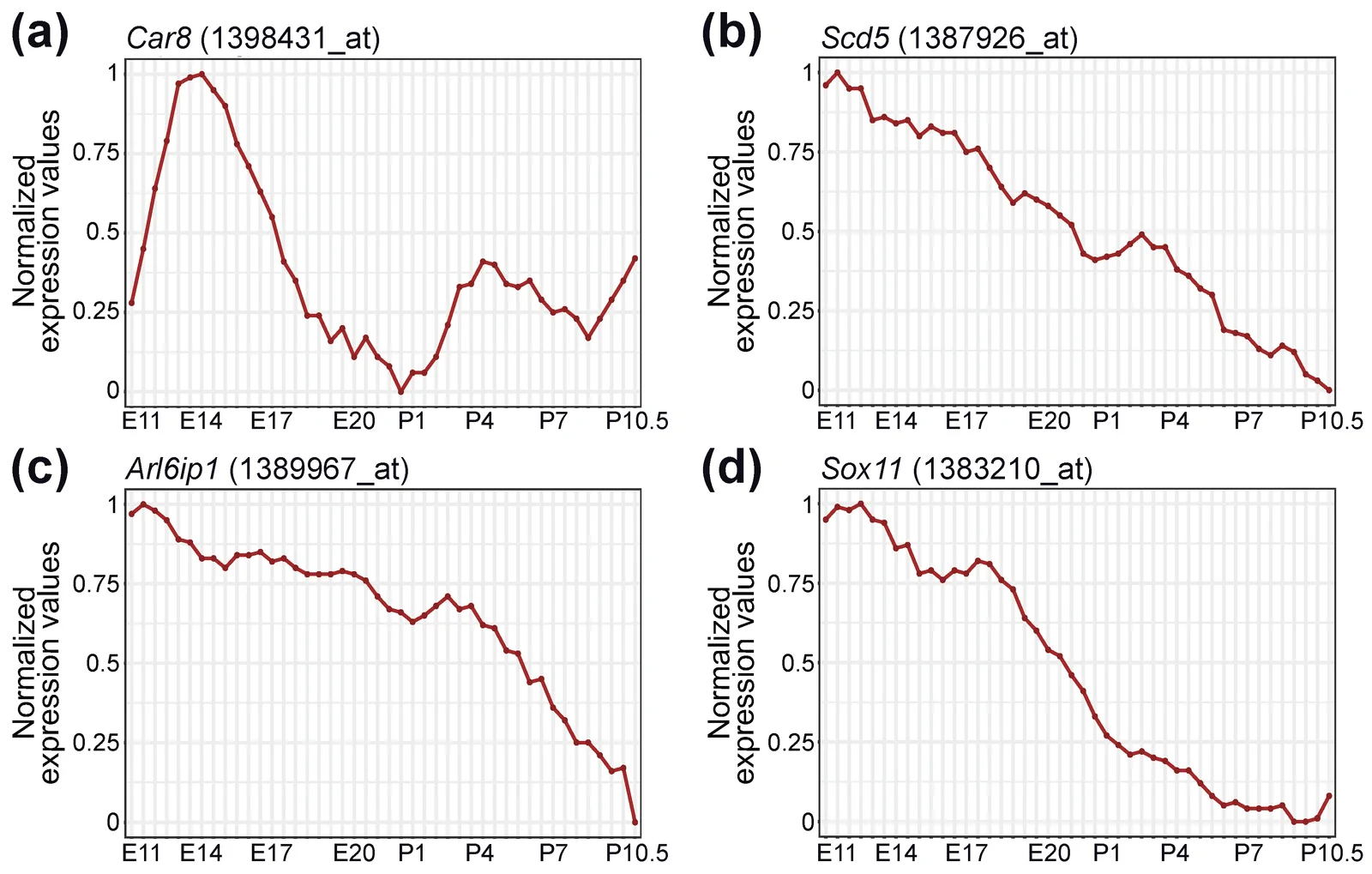
Expression profiles of novel candidate cytokinesis genes chosen for experimental validation. (**a**–**d**) Gene expression profiles of the four experimentally validated genes (i.e., Car8, Scd5, Arl6ip1, and Sox11) in the heart development dataset across the 44 time points.

**Figure 5:**
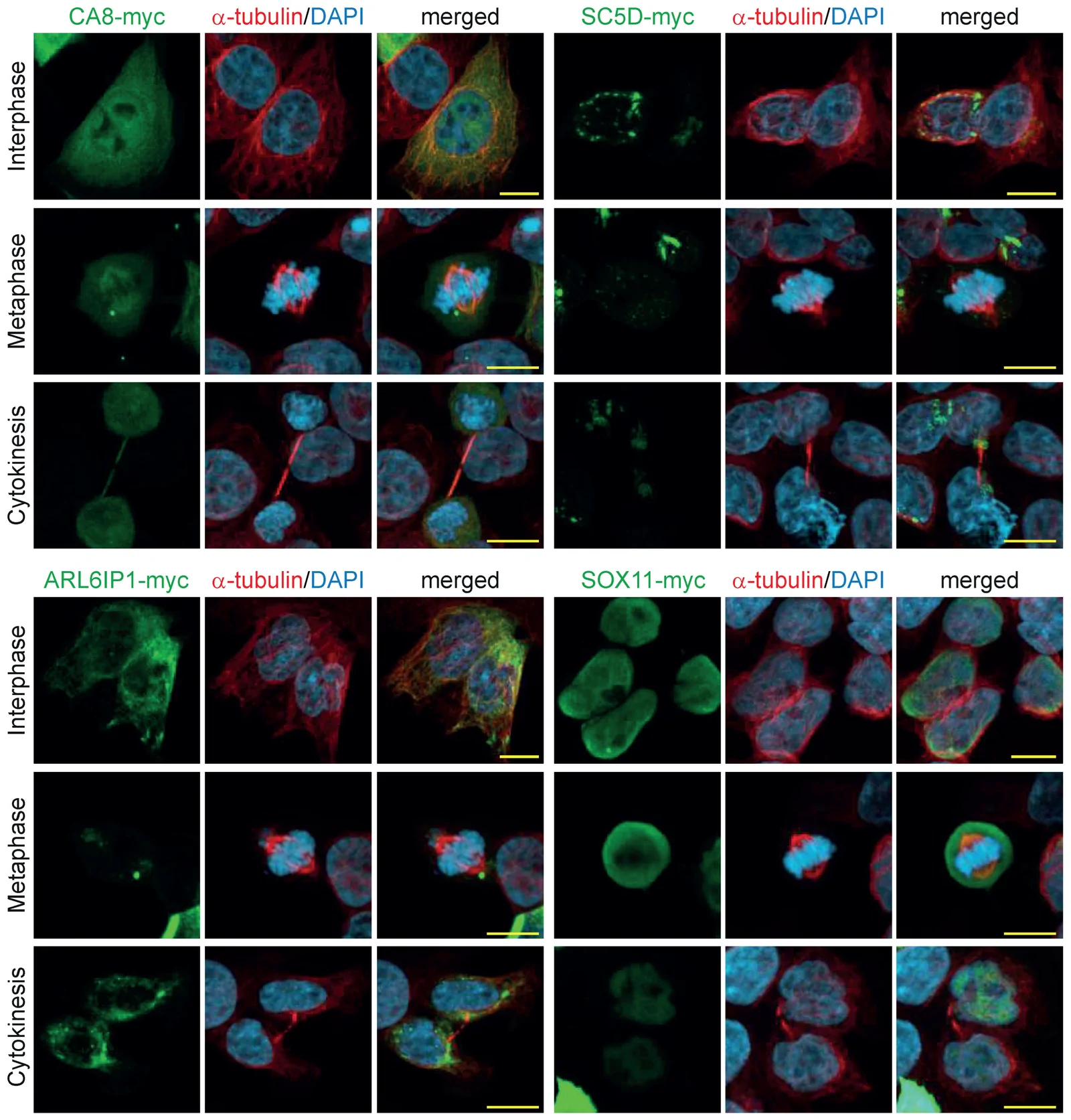
Localization of CA8, SC5D, ARL6IP1 and SOX11 during mitosis. HeLa cells were transfected with CA8, SC5D, ARL6IP1 and SOX11 tagged with myc and subsequently stained for myc (green) and α-tubulin (red). Nuclei were visualized by DAPI (blue). Scale bars: 10 µm.

**Figure 6:**
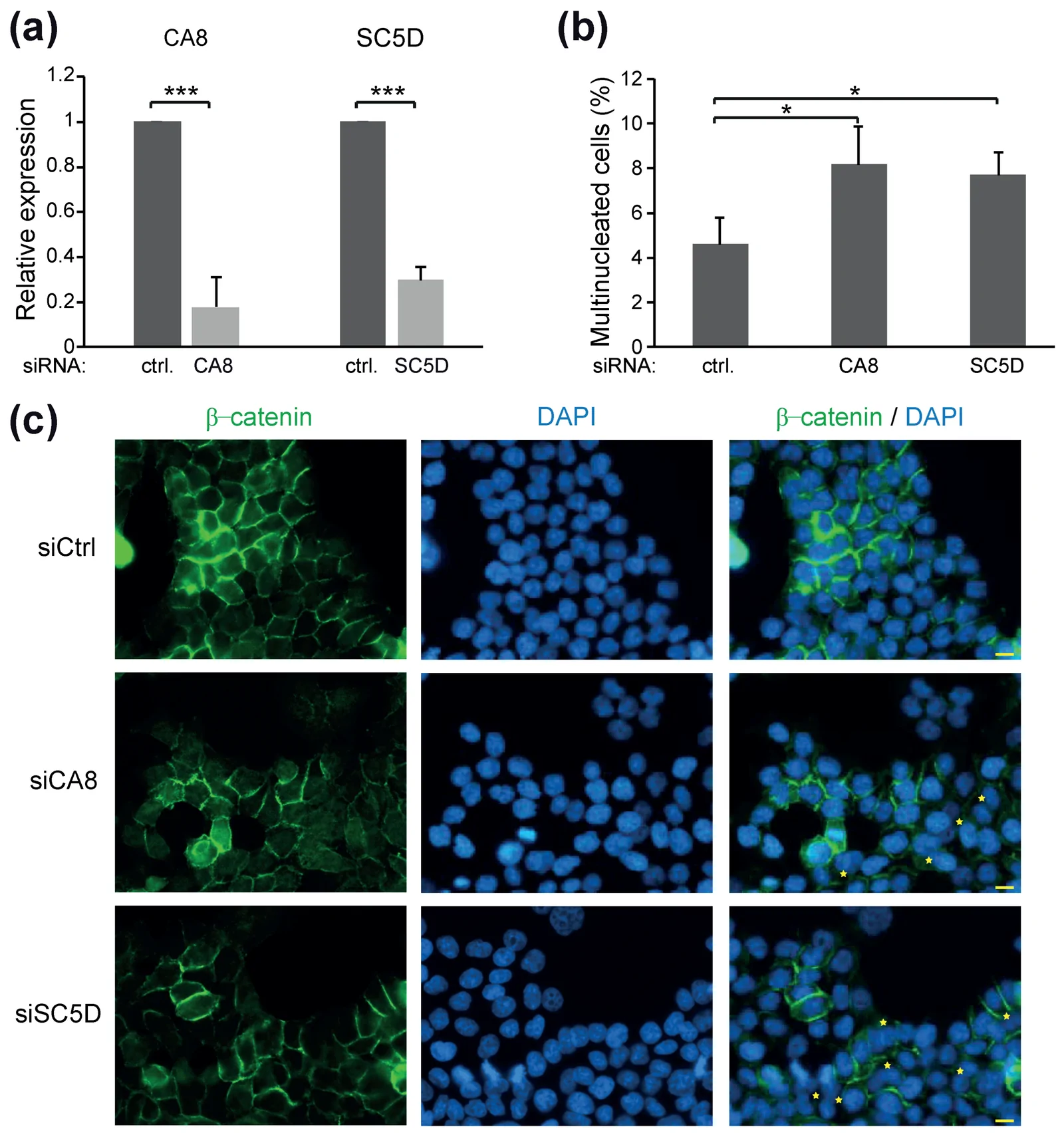
Knockdown of CA8 and SC5D in Hct116 cells. Hct116 cells were transfected with a control siRNA (ctrl.), CA8-, or SC5D-specific siRNAs. (**a**) Knockdown efficiency measured by RT-PCR. Data are mean ± S.D. ***: *p*-value ≤ 0.001 (**b**) Percentage of multinucleated Hct116 cells was quantified 72 h post-transfection. *n* > 343 cells from three independent experiments. Data are mean ± S.D. *: *p*-value ≤ 0.05. (**c**) Representative images of Hct116 cells transfected with siCtrl, siCA8, or siSC5D showing the presence of multinucleated cells (yellow asterisks). The staining for β-catenin (green) allows discriminating the cell membrane. Scale bars: 10 µm.

## Data Availability

The data supporting the findings of this study are available within the paper and its Supplementary Information.

## Supplementary Materials

Supporting information can be downloaded at: https://www.mdpi.com/article/10.3390/ijms27198459/s1.

## Abbreviations

Cy3	cyanine 3
Cy5	cyanine 5
DE	differentially expressed
DSMO	dimethyl sulfoxide
E	embryonic
FC	fold change
FGF1	fibroblast growth factor 1
GO	Gene Ontology
HD	heart development
KO	knockout
LH	list hit
LT	list total
MEF	mouse embryonic fibroblast
P	postnatal
p38i	p38 inhibitor
PH	population hits
PT	population total
RNA	ribonucleic acid
WT	wild type
