# Supplementary material for: Integrative Bioinformatics Across Proliferative and Non-Proliferative Cell States Reveals Novel Cytokinesis Genes
Source: Int J Mol Sci. 2026 Sep 23;27(19):8459. doi: 10.3390/ijms27198459 (PMC13664727; doi:10.3390/ijms27198459)
Supplement: Supplementary file 1 [file ijms-27-08459-s001.zip › AngeloniM_SuppFigures.pdf]

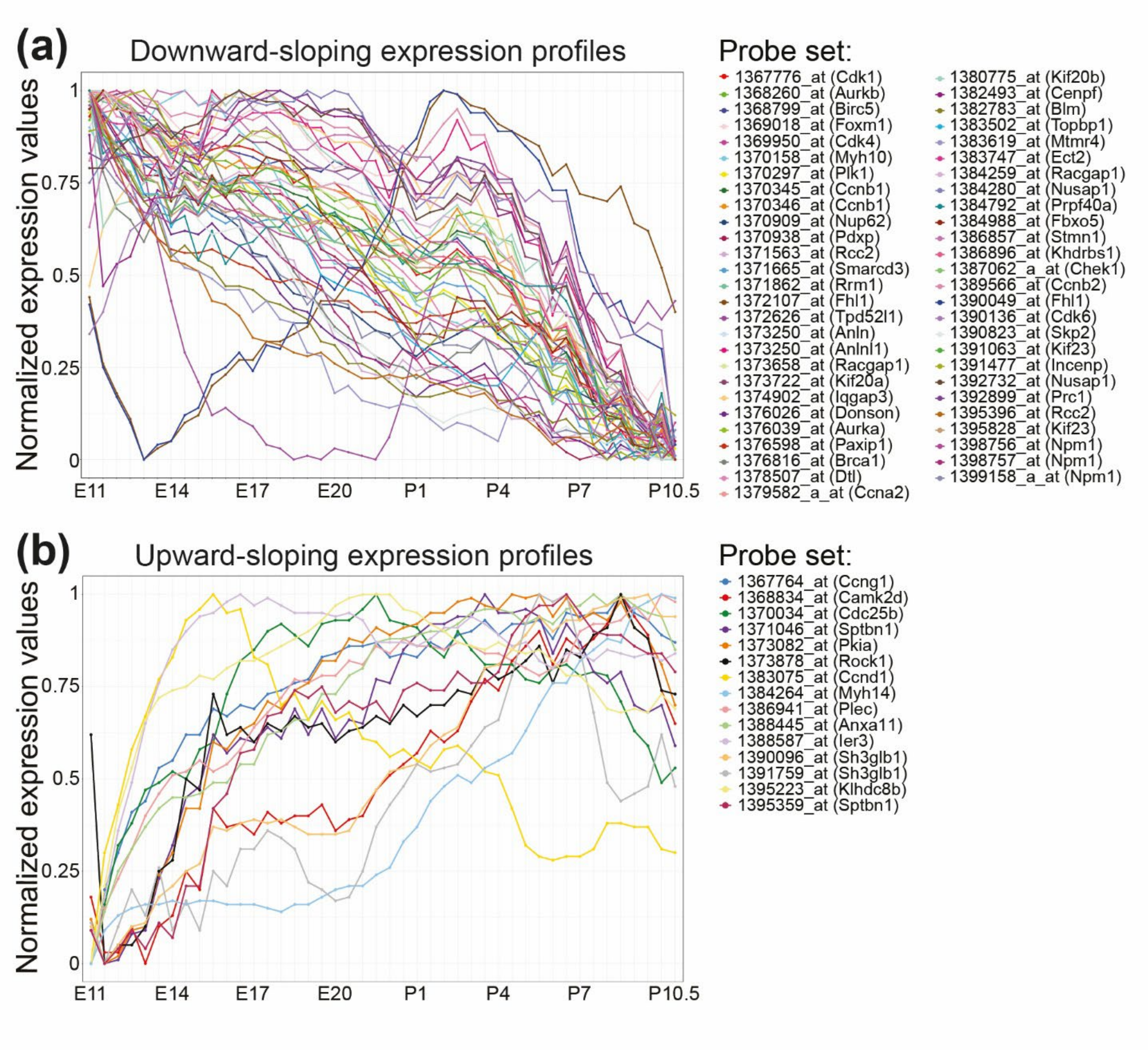

Figure S1. Expression profiles of GO-based cytokinesis-related genes in the HD dataset. Gene expression profiles of the differentially expressed probe sets in the heart development dataset mapping to GO-based cytokinesis-related genes. The probe sets are divided into those with **(a)** downward- and **(b)** upward-sloping profiles. Profiles were categorized as downward if the normalized expression value of the first time point was higher than the normalized expression value of the last time point, upwards otherwise. The gene symbol associated with a given probe set is provided in parenthesis. E: embryonic; P: postnatal.

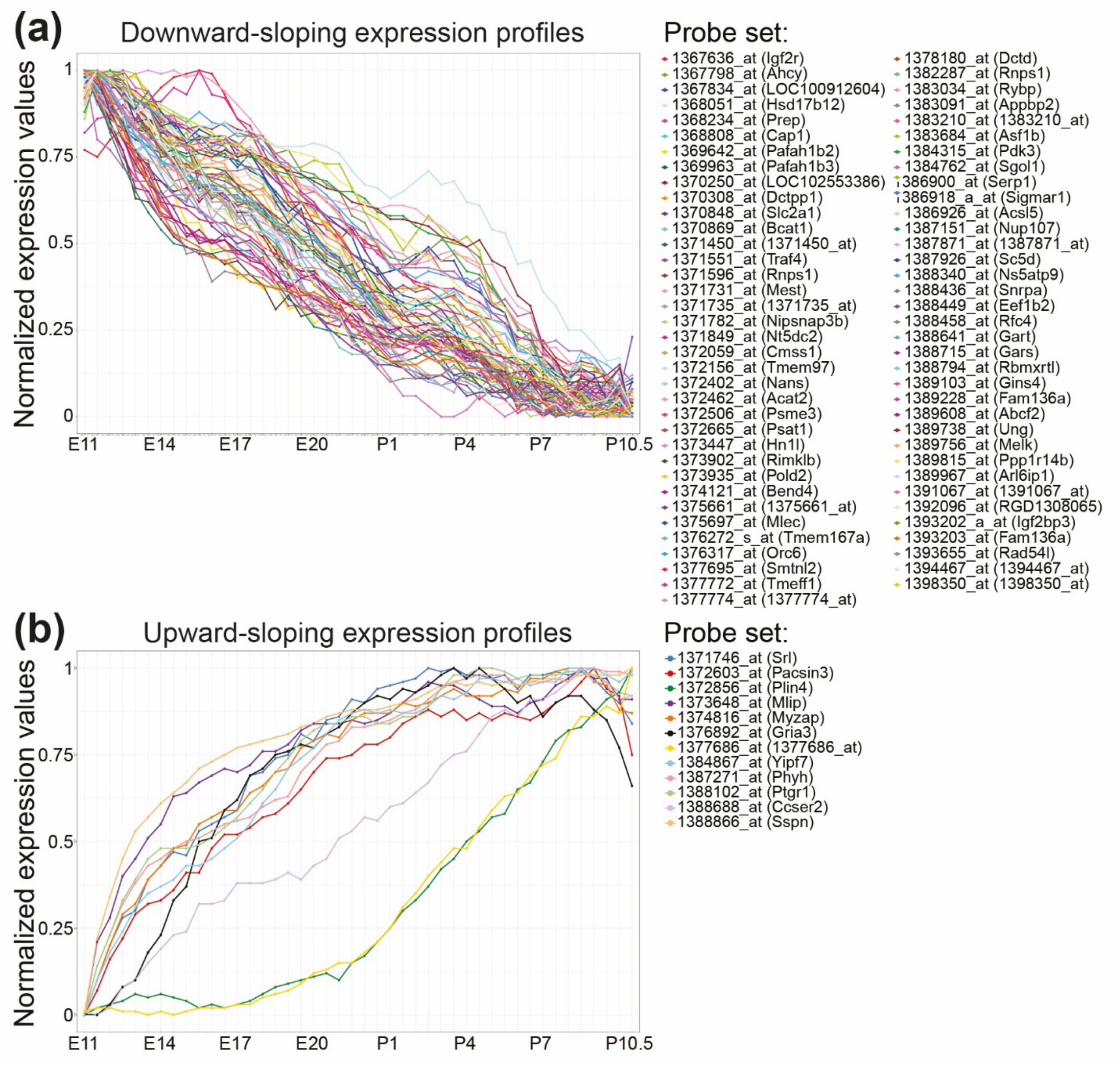

Figure S2. Expression profiles of the knowledge-driven candidate cytokinesis genes. Gene expression profiles of the knowledge-driven candidate cytokinesis genes. The genes are divided into genes with **(a)** downward- and **(b)** upward-sloping profiles. Profiles were categorized as downward if the normalized expression value of the first time point was higher than the normalized expression value of the last time point, upwards otherwise. E: embryonic; P: postnatal.

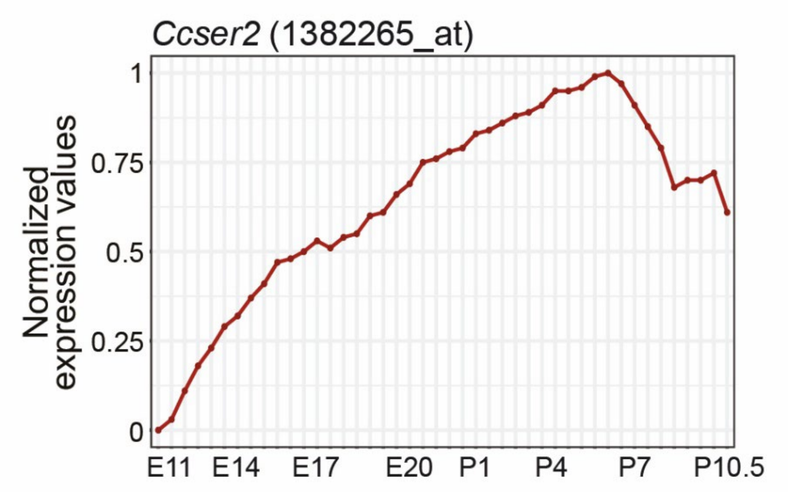

Figure S3. *Ccser2* expression profile. Normalized gene expression profile of *Ccser2* in the heart development dataset. E: embryonic; P: postnatal.
