# Supplementary material for: Integrative Bioinformatics Across Proliferative and Non-Proliferative Cell States Reveals Novel Cytokinesis Genes
Source: Int J Mol Sci. 2026 Sep 23;27(19):8459. doi: 10.3390/ijms27198459 (PMC13664727; doi:10.3390/ijms27198459)
Supplement: Supplementary file 1 [file ijms-27-08459-s001.zip › AngeloniM_SuppTable_legends.pdf]

Supplementary Table Legends

Table S1. Mouse gene symbols to rat orthologues mapping. For each mouse gene symbol (column: "Mm gene symbol") the table reports the associated spot(s) (column: "Spot(s)") on the Agilent Whole Mouse Genome Microarray 4x44K as well as the corresponding rat orthologue gene symbol (column: "Rn orthologue"), when available. In case of multiple spots available for a given gene, one representative spot was selected based on the lowest coefficient of variation (column: "Chosen spot"). Mm = Mus musculus; NA = not available; Rn = Rattus norvegicus.

Table S2. Cytokinesis-related GO terms. In the sheet "GO_All_Primary_Matches" all the GO terms, including the obsolete ones, matching the regular expression "cytokine[^\\s-]|m phase" are reported. For each GO term, the associated identifier (column: 'GO_ID'), name (column: 'GO_NAME'), and description (column: 'GO_DESCRIPTION') are provided. The sheet "GO_Final_Primary_Matches" reports all the primary matches excluding the obsolete terms. All the child terms of the final primary matches are listed in the sheet "GO_Child_Terms". The final cytokinesis-related terms, with the number (column: 'n_genes') and list (column: 'genes') of associated genes, are reported in the sheet "GO_Final_Cytokinesis_Terms". NA = not available.

Table S3. GO-based cytokinesis-related genes. The table reports the final list of 326 genes annotated to cytokinesis-related processes in the Gene Ontology (GO).

Table S4. Cell cycle-related GO terms. In the sheet "GO_All_Primary_Matches" all the GO terms, including the obsolete ones, annotated with one of the following words or regular expression: "cell cycle", "spindle", "mitotic", "cell differentia", "heart development", "cell division", (cytokine[^\s-]), "m phase" are reported. For each GO term, the associated identifier (column: 'GO_ID'), name (column: 'GO_NAME'), and description (column: 'GO_DESCRIPTION') are provided. The sheet "GO_Final_Primary_Matches" reports all the primary matches excluding the obsolete terms. All the child terms of the final primary matches are listed in the sheet "GO_Child_Terms". The final cell cycle-related terms, with the number (column: 'n_genes') and list (column: 'genes') of associated genes, are reported in the sheet "GO_Final_CellCyle_Terms". NA = not available.

Table S5. GO-based cell cycle-related genes. The table reports the final list of 6049 genes annotated with cell cycle-related processes in the Gene Ontology (GO).

Table S6. Knowledge driven candidate cytokinesis genes. The table reports, for each knowledge driven candidate gene, the probe set (column: "Probe set") as well as the corresponding gene symbol (column: "Gene identifier"). If for a probe set the corresponding gene symbol is missing, the probe set is repeated in the column "Gene identifier".

Table S7. p38i+FGF1 candidate cytokinesis genes. The table reports, for each candidate cytokinesis gene, the probe set (column: "Probe set") as well as the corresponding gene symbol (column: "Gene identifier"). If for a probe set the corresponding gene symbol is missing, the probe set is repeated in the column "Gene identifier".

Table S8. Lin9 candidate cytokinesis genes. The table reports, for each Lin9 candidate cytokinesis gene, the gene identifier (column: "Gene identifier") as well as the corresponding spot in the Agilent Whole Mouse Genome Microarray 4x44K (column: "Spot (Mouse Array)") and corresponding probe set(s) in the Affymetrix RAE230 array (column: "Probe set (Affymetrix RAE230)"). When multiple probe sets are associated with the same gene identifier, they are all reported and separated by "//".

Table S9. Final list of candidate cytokinesis genes. The sheet "final_candidates_genes" reports, for each of the 236 final candidate cytokinesis genes, the probe set(s) in the Affymetrix RAE230 array (column: "PROBE_SET_RAE"), the corresponding spot in the Agilent Whole Mouse Genome Microarray 4x44K (column: "SPOT_LIN9"), as well as the

corresponding gene symbol (column: “GENE_IDENTIFIER”). If for a probe set the corresponding gene symbol is missing, the probe set is repeated in the column “GENE_IDENTIFIER”. The sheet “prioritized_candidate_genes” reports for each of the 15 prioritized genes the probe set(s) in the Affymetrix RAE230 array (column: “PROBE_SET_RAE”), the corresponding gene symbol (column: “GENE_IDENTIFIER”), as well as the corresponding spot in the Agilent Whole Mouse Genome Microarray 4x44K (column: “SPOT_LIN9”). If for a probe set the corresponding gene symbol is missing, the probe set is repeated in the column “GENE_IDENTIFIER”. The column “GENE_SYMBOL_BLASTING” shows the mapped gene symbol obtained by blasting of the target sequence. HD = heart development; DE = differentially expressed.
